# Multiphysical Field Modulated VO_2_ Device for Information Encryption

**DOI:** 10.1002/advs.202300908

**Published:** 2023-04-28

**Authors:** Shanguang Zhao, Liang Li, Changlong Hu, Bowen Li, Meiling Liu, Jinglin Zhu, Ting Zhou, Weidong Shi, Chongwen Zou

**Affiliations:** ^1^ National Synchrotron Radiation Laboratory School of Nuclear Science and Technology University of Science and Technology of China Hefei Anhui 230029 P. R. China; ^2^ Research Institute of Chemical Defense Beijing 102205 P. R. China

**Keywords:** encryption, phase transition, vanadium dioxide

## Abstract

In the information explosion society, information security is highly demanded in the practical application, which raised a surge of interest in designing secure and reliable information transmission channels based on the inherent properties of emerging devices. Here, an innovative strategy to achieve the data encryption and reading during the data confidential transmission based on VO_2_ device is proposed. Owing to the specific insulator‐to‐metal transition property of VO_2_, the phase transitions between the insulator and metallic states are modulated by the combination of electric field, temperature, and light radiation. These external stimulus‐induced phase diagram is directly associated with the defined VO_2_ device, which are applicable for control the “0” or “1” electrical logic state for the information encryption. A prototype device is fabricated on an epitaxial VO_2_ film, which displayed a unique data encryption function with excellent stability. The current study not only demonstrated a multiphysical field‐modulated VO_2_ device for information encryption, but also supplied some clues for functional devices applications in other correlated oxide materials.

## Graphical abstract

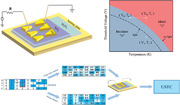

## 1 Introduction

With the development of information technology, information security is one of the key issues in the fields of politics, military, economy, and daily life, which makes the information encryption/decryption technology extremely important and attracts wide attention.^[^ ^1^, ^2^, ^3^, ^4^ ^]^ The commonly used information security techniques include watermarking,^[^ ^5^, ^6^ ^]^ security inks,^[^ ^7^, ^8^, ^9^ ^]^ fluorescent identification,^[^ ^10^, ^11^ ^]^ and graphical barcodes.^[^ ^12^, ^13^ ^]^ While currently, materials that respond to external chemical and physical stimuli show great application prospects in information encryption technology.^[^ ^14^ ^]^ For example, polymer gels will produce color changes under external light,^[^ ^15^ ^]^ heat,^[^ ^16^ ^]^ electricity,^[^ ^17^, ^18^ ^]^ mechanical force,^[^ ^19^ ^]^ or other stimuli, which can be used as anti‐counterfeit labels by the color changes to the naked eyes. However, this kind of encryption technique is likely to be copied or imitated because the color change is an easily observed output that can be brute‐force by analyzing stimulus‐response models.

As a typical correlated material, VO_2_ crystal undergoes significant insulator‐to‐metal transition (IMT) behavior near 340K, accompanied by great changes in conductivity, infrared transmittance, and other properties.^[^ ^20^, ^21^, ^22^ ^]^ This unique IMT behavior of VO_2_ compound makes it broad applications in the fields of ultrafast photoelectric switching,^[^ ^23^ ^]^ neuron computing,^[^ ^24^, ^25^ ^]^ resistive storage,^[^ ^26^ ^]^ microbrakes,^[^ ^27^ ^]^ etc. Importantly, the insulator‐metal phase transition of VO_2_ can be effectively triggered or modulated by various external stimuli such as elemental doping, temperature, electric field, magnetic field, light, and stress.^[^ ^28^, ^29^, ^30^, ^31^, ^32^, ^33^, ^34^, ^35^, ^36^, ^37^ ^]^ Among them, the temperature, light, and electric field can reversibly modulate the phase transition of VO_2_ and be easily manipulated, which is applicable for different functional devices via the phase transition.

In the current study, we propose an information encryption strategy based on the phase transition diagram of VO_2_ device upon the multiple stimuli including the bias voltage, temperature, and light radiation, which are adopted for the separated encryption channels. By using these multichannels stimulus, the original binary information encoding sequence can be transferred to the combinations of temperature, light, and electric field timing codes. The information receiver will read the multichannel information and decode the original information according to the phase transition diagram of VO_2_ device. This encryption strategy will greatly reduce the risk of information being intercepted and deciphered, showing important application prospects based on this multiphysical field‐modulated VO_2_ device in the future.

## 2 Results and Discussion

### 2.1 Characterizations of the Epitaxial VO_2_ film

**Figure 1:**
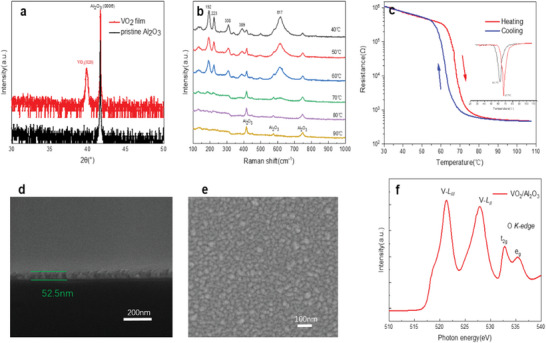
Characterization of VO_2_/ Al_2_O_3_ (0001) thin film. a) XRD characterizations for the film sample. b) Temperature‐dependent Raman spectroscopy for VO_2_ thin film. c) The *R–T* curve for VO_2_ thin‐film. The differential curve was plotted in the inset, showing two critical temperatures during the test loop. d) SEM cross‐section of VO_2_ thin film deposited on Al_2_O_3_. The thickness of VO_2_ thin film is ≈52.5 nm. e) SEM images for the surface morphology. f) The XANES curve for the prepared VO_2_ film.

The epitaxial M‐VO_2_(020) films were grown on Al_2_O_3_(0001) through oxide molecular beam epitaxy (O‐MBE), determined by the sharp peak at 39.8° in X‐ray diffraction (XRD) patterns (**Figure** **1**a). This observation shows that the VO_2_ films were grown with the preferred orientation. The structure information of VO_2_ films was further examined by temperature‐dependent Raman measurements (Figure 1b). It showed that the strong Raman peaks at 192, 223, 308, 389, and 617 cm^−1^ were observed at room temperature, confirming the presence of monoclinic VO_2_ films by the oxide molecularbeam epitaxy.^[^ ^38^, ^39^ ^]^ As the temperature increasing, the Raman peak of monoclinic VO_2_ disappears gradually, indicating the temperature‐driven phase transformation of VO_2_ films from monoclinic to rutile phase. When the temperature rises to 90 °C, only the Raman peak of the Al_2_O_3_ substrate can be observed.

To evaluate the phase transition properties of VO_2_ thin films, the temperature‐dependent electrical resistance of the resultant VO_2_ films was measured by a four‐probe measurement system. Figure 1c shows an apparent resistance phase transition of three orders of magnitude from the high resistance state to the metallic state for the VO_2_ samples. According to the differential curve in the insert, it can be observed that the phase transition temperature in the heating process is 67.7 °C, and that in the cooling process is 62.3 °C, which is very consistent with the previous report.^[^ ^40^, ^41^ ^]^

Figure 1d shows the SEM cross‐section of the film, where a distinct difference between the VO_2_ film and the alumina substrate can be observed. The Surface topography of the VO_2_ sample was also examined by SEM and AFM as shown in Figure 1e and Figure S1, Supporting Information. It can be observed that the surface of VO_2_/ Al_2_O_3_ sample is compact and smooth, and the distribution of nanograins is uniform. AFM surface morphology characterization further shows that smooth and flat vanadium dioxide films were obtained in Figure S2, Supporting Information. To further examine the chemical states of VO_2_ films, XANES characterization based on synchrotron radiation was shown in Figure 1f. The XANES curve shows the clear V‐L edge at 528.0 eV (2p_1/2_) and 521.4 eV (2p_3/2_) as well as the O‐K edge with the *e*_g_ and *t*_2g_ peaks. According to the energy position of the V‐L edges and the O‐K edge peaks, we can further confirm the VO_2_ compound in the prepared film.^[^ ^42^ ^]^

**Figure 2:**
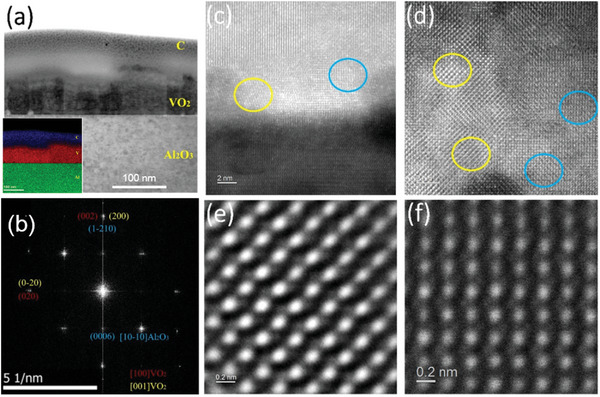
HRTEM characterization of epitaxial VO_2_ film. a) Low‐magnification HAADF image taken by STEM showing both orientations growing columnar along [10‐10] sapphire zone axis. The insert shows the EDS mapping for the epitaxial VO_2_ sample on Al_2_O_3_ (0001) substrate. b) The diffraction pattern belongs to both crystallites of VO_2_ along [001] and [100] and [10‐10] substrate; c) HAADF image taken from the interface between VO_2_ and sapphire substrate. The dark region represents the lattice phase of sapphire and two sets of VO_2_ domains (marked with blue or yellow circles) can be observed at the interface. d) HAADF‐STEM image shows that there are two sets of VO_2_ domains contained within the epitaxial sample. One colored circle indicates the corresponding set of domains. e) STEM‐HAADF high‐resolution image of bright region in (d) (yellow circles) and f) HAADF image of dark region in (d) (blue circles).

To further confirm the component and the quality of the crystal VO_2_/c‐sapphire thin‐film heterostructure, the high angle annular dark field images (HAADF) and selected area electron diffraction patterns (SAED) were conducted by the scanning transmission electron microscope (STEM). **Figure** **2**a represents the low‐magnification HAADF image along [10‐10] sapphire zone axis. The related energy dispersive spectroscopy (EDS) image in the insert of Figure 2a illustrates the clear heterogeneous interface between VO_2_ film and sapphire substrate, the precise distribution of V and Al elements. Figure 2b shows the diffraction pattern belong to both crystallites of VO_2_ along [001] and [100] and **[10‐10]** sapphire substrate. The high‐resolution HAADF images exhibit an alternate stacking of bright and dark regions along the substrate surface in the VO_2_ layer, as illustrated by different colored circles in Figure 2c,d. The alternate stack and uniformity of lattice spacing confirm the excellent crystallinity. Detailed investigation of bright and dark regions is shown in Figure 2e,f, respectively, where Zhou et al. have indexed the image of Figure 2e to the [100] direction of VO_2_ and Figure 2f to VO_2_[001] or VO_2_[120].^[^ ^43^ ^]^ Accordingly, from the STEM results, it can be observed that there really exist some dislocations and grain boundaries in the films as shown in Figure 2c,d, which is due to the epitaxial film growth and the distinct crystal structure symmetry of VO_2_. While on the one hand the atomic resolved STEM‐HAADF images, it was confirmed the excellent crystal quality of VO_2_ epitaxial film.

### 2.2 Fabrication and Electric Property of VO_2_ Device

**Figure 3:**
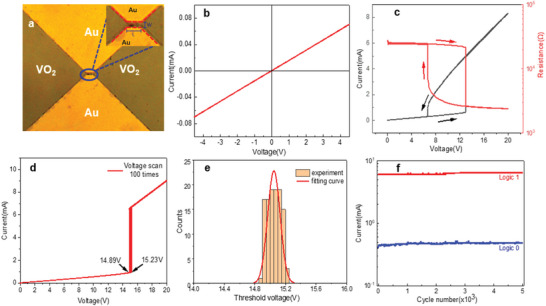
Electrical measurement of VO_2_ thin films. a) The optical photograph of the VO_2_ film with the Au‐electrodes. The definition of the gap dimension was shown in the insert. b) The linear *I–V* curve confirms the good Ohmic contact. c) The *V–I* and *V–R* curves measured at 40 °C. d) The voltage scans from 0 V to 20 V with 100 times for the repeatability test. e) The threshold voltage distribution and fitting curve for these 100‐time voltage scans. f) The voltage‐driven logic “0” and “1” states with the cycle of 5000 times.

The electrodes with different gap sizes were fabricated by UV lithography, and the detailed process was described in Figure S3. The optical photograph for a typical VO_2_ film with Au‐electrodes was shown in **Figure** **3**a. The spacing between electrodes on the micron scale can be clearly observed. In order to examine the Ohmic contact of the prepared electrodes, the typical *I–V* measurement is conducted, as shown in Figure 3b. The voltage of the device is scanned forward and reverse, a linear *I–V* curve is obtained, confirming the good Ohmic contact between the electrode and the film.

It has been reported that VO_2_ will also undergo a phase transition from insulation M1 to metal R phase under the action of an electric field, which is generally considered to be induced by electric field and electric heating effect.^[^ ^44^ ^]^ Due to the first‐order phase transition of VO_2_,^[^ ^45^ ^]^ its resistance and current will jump under the action of electric field. The insulation state can be read as “0” state, and the metal state after the current jump can be read as “1” state for electronic logic devices.^[^ ^46^ ^]^ Figure 3c shows the V–I and *V–R* curves, measured with increasing and then decreasing the applied voltage at temperature of 40 °C. When the applied voltage increases slowly from zero, the current increases monotonically. When the voltage reaches the phase transition threshold voltage *V*_IMT_, the current suddenly jumps to a higher value, accompanied by a drop‐down of resistance value. When scanning from high voltage to low voltage in a reverse way, the current decreases slowly almost linearly. Once the voltage reaches the other *V*_IMT_, the current value decreases suddenly and the device goes back to the initial insulate state.

For electronic devices, the consistency between different *I–V* testing cycles is particularly important, which is mainly determined by the quality of the VO_2_ film. According to previous studies, the phase transition threshold voltage of vanadium dioxide films is affected by many factors, so it is crucial to obtain high‐quality films with stable phase transition threshold voltage for their applications in electronic devices. Figure 3d depicts 100 voltage sweep cycles from 0 V to 20 v, demonstrating extremely stable threshold switching and low cycle‐to‐cycle variation. Within these 100 cycles, the minimum switching threshold voltage is 14.89 V and the maximum is 15.23 V. Figure 3e shows that in the threshold voltage distribution of 100 cycles, the number is mainly concentrated ≈15 V, and the smallest part of the value deviates from 15 V by no more than 0.2 V. The resistance conversion in VO_2_ is attributed to the intrinsic electronic and structural phase transition of the material itself, so the low threshold voltage change can be attributed to the high crystalline quality of epitaxial VO_2_.

Owing to the sharp phase transition of VO_2,_ its insulate and metallic states can be read and written as “0” and “1” logic state, which can be triggered by the external voltage or electric field. Figure 3f demonstrates that controlled by the electric field, the prepared VO_2_ film device can be reliably read 5000 times “0” and “1” without performance deterioration. In addition, the transient electrical measurements shows that the switching speed of the device is <120 µs from off‐state to on‐state (Figure S4, Supporting Information) by this voltage sweeping mode.

### 2.3 The Phase Diagram of VO_2_ Device

**Figure 4:**
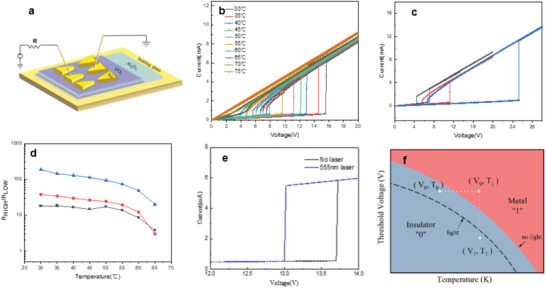
The phase transition of VO_2_ device is controlled by temperature and electric field. a) Scheme for the two‐terminal VO_2_ device. b) The temperature‐dependent switching voltage for the VO_2_ device with the spacing size of 18 µm x 5 µm. c) The switching threshold voltages measured at 50 °C for the VO_2_ device with the spacing size of 22 µm x 10 µm (blue curve), 18 µm x 5 µm (red curve), and 14 µm x 2 µm (black curve), respectively. d)The resistance ratio (*R*_High_/*R*_Low_) values for the VO_2_ device with different electrode spacing sizes; e) The *I–V* curve of the VO_2_ device with or without the light radiation, showing different threshold voltage values; f) The phase diagram of VO_2_ device modulated by multiphysical fields.

**Figure** **4**a shows a schematic diagram of the experiment on a temperature‐controlled stage. During the test, the I‐V curves can be examined at different temperatures. Since the resistance of metallic VO_2_ film after phase transition is quite low, the circuit is protected by an external resistor. Figure 4b shows the temperature‐dependent switching voltage of the VO_2_ device with the electrode spacing gap of ≈18 × 5 µm. The curve shows that the switching voltage of VO_2_ film decreases with the increase of temperature, and it still shows obvious switching change until 65 °C, which is close to the phase transition temperature. This is consistent with the previous *R‐T* curve. It is observed that the electrode spacing gap size directly determines the switching threshold voltage value as shown in Figures S5–S6, Supporting Information, and Figure 4c. Normally, the bigger gap size, the larger switching threshold voltage is required to trigger the phase transition as shown in Figure 4c. From this figure, it is obvious that the phase transition threshold voltage increases with the electrode gap of the device, which is considered to be due to the larger electrode gap requires more heat accumulation.^[^ ^47^ ^]^

Figure 4d shows the resistance jump ratio (*R*_High_/*R*_Low_) at the threshold voltage *V*_IMT_ point (for the increasing voltage scan process) for the prepared VO_2_ devices at different temperatures. For the device with the electrode gap sizes of 22 × 10 µm (the blue triangles and blue line), it is clear that the device has the largest switching resistance ratio (*R*_High_/*R*_Low_) values up to more than two orders of magnitude at 30 °C, while this ratio value will decrease with the temperature increasing. For comparison, the VO_2_ devices with the electrode gap sizes of 18 × 5 µm and 14 × 2 µm are also tested as shown in the figure (the red dots and black cubes), respectively. It can be observed that all of the devices show decreased resistance ratio (*R*_High_/*R*_Low_) values as the temperature increasing. While it should be noticed that even close to the critical phase transition temperature (e.g., at ≈65 °C), the resistance ratio of *R*_High_/*R*_Low_ values are still larger than one order of magnitude, showing excellent contrast between the “0” or “1” logic states for practical device application.

Furthermore, the electric field‐driven phase transition process of VO_2_ devices can also be modulated by external light radiation. Figure 4e shows the *I–V* curves for the VO_2_ device during the increasing sweeping voltage with or without the radiation of 555 nm laser light. During the measurement, the sample stage is kept a constant temperature of 40 °C. It was clear that without the laser radiation, the threshold voltage is ≈13.6 V. While if the laser radiation is turned on, the voltage will be decreased to 13.0 V. In fact, other laser radiation with different wavelengths also indicates that the light radiation will change the threshold voltage for this VO_2_ device as shown in Figure S7, Supporting Information. Therefore, suitable light radiation can also be used as an external stimulus to regulate the VO_2_ phase transition, which is quite consistent with previous reports.^[^ ^48^, ^49^, ^50^ ^]^

According to the above experimental results, we are able to plot the phase diagram for the VO_2_ device as shown in Figure 4f. Since the specific switching threshold voltage value is determined by the device temperature, we can obtain two areas with different colors, which are related to the insulator or metallic state, or the “0” and “1” logic state. Thus different (*V, T*) serials will determine the final phase structure of VO_2_ device, or determine the output result to be “0” or “1” logic state. For example, as the voltage is V_0_ and the temperature is *T*_1_, the output result is “1”, which is related to the point (*V*_0_, *T*_1_) in the phase diagram. While for the same device, if keeping the temperature unchanged at *T*_1_, while decreasing the voltage from *V*_0_ to *V*_1_, the output will change to “0”, as showing by the point (*V*_1_, *T*_1_) in the phase diagram. Furthermore, if adding light radiation as the third parameter to control the phase transition process, the phase boundary will be moved as shown by the dashed line in Figure 4f.

### 2.4 Multichannel Information Encryption

**Figure 5:**
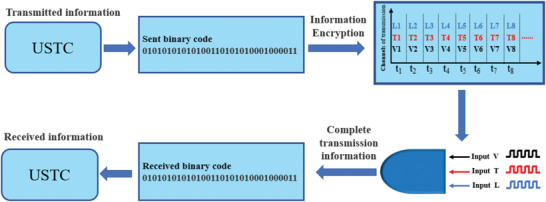
Schematic diagram of multichannel physical field stimulus information encryption and transmission. The “USTC” letters related binary codes will be encrypted by the VO_2_ device with the three channels including the light radiation (*L*), temperature (*T*) and voltage (*V*) stimulus. According to the hardware‐dependent VO_2_ device, the receiver will decrypt the data from the three channels and obtain the original information.

Based on the phase transition of VO_2_ material, R. F. Haglund group proposed a multilevel, multicolor metasurface encryption device with the two decryption keys of temperature and hydrogenation.^[^ ^36^ ^]^ While this encryption system was mainly lying on the four states of the optical display controlled by the temperature and H‐doping process, which made it slow response, complex fabrication, and low system integration. While according to the above phase diagram of the VO_2_ device driven by different external stimuli in Figure 4f, we can achieve a multichannel information encryption conveniently for practical application. The basic principle for the information encryption device by multiple physical field channels is shown in **Figure** **5**. The information (e.g.,“USTC” letters) is transformed into binary codes. For each bit of code, it is converted to the output result of the VO_2_ device controlled by multiple physical fields, including voltage, temperature, and light excitation. The applied physical field is binary encoded and transmitted through three independent channels. After that, the transmission information is obtained by applying voltage, temperature, and light radiation parameters to the same VO_2_ device. This method of compiling data into multiple physical field data and transmitting it over multiple channels greatly reduces the risk of data being deciphered. If the invader uses incomplete channel parameters to read information, misleading results will be obtained. More importantly, even if all three information channels of transmission are intercepted, the information cannot be accurately deciphered due to the different inherent parameters in the prepared VO_2_ device. That is to say, this encryption and decryption process are both hardware dependent, thus the accurate information can only be obtained by combining the transmission data of different channels on the unique VO_2_ device, which has the unique phase diagram as shown in Figure 4f.

**Figure 6:**
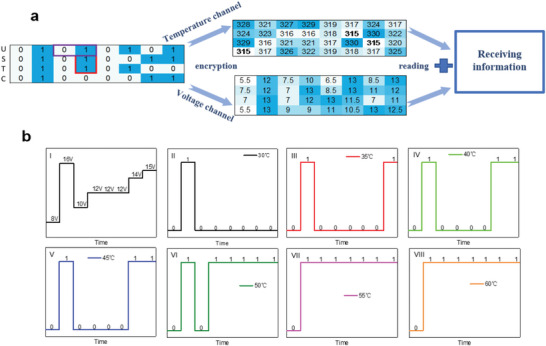
Demonstration of information encryption stimulated by temperature‐electric field dual physical fields**. a)** The original information will be transferred by the temperature and voltage channels for encryption. Then the receiver will decrypt the information by the VO_2_ device. **b**) The two channels are closely correlated together for the performance of this VO_2_ device according to the external field‐triggered phase diagram. For example, with the same voltage serials in Figure‐I, different data will be obtained under different temperatures from 30 °C to 60 °C, as shown in Figure‐II to Figure V‐III, confirming the one‐way encryption.

According to the phase diagram as shown in Figure 4f, we demonstrate a two‐channel information encryption process, which includes the channel of temperature and channel of voltage. As shown in **Figure** **6**a, the “USTC” letters related binary serials including the “0” and “1” logic states can be encrypted by the temperature channel and voltage channel with different temperature values or voltage values. For example, for the first “0” logic state, it is related to (328, 5.5), since according to the phase diagram, the VO_2_ device will be an insulator state when the temperature is at 328K and the voltage is set at 5.5 V. While for the second “1” logic state, it is related to (321, 12), thus it will be a metallic state according to the phase diagram of the device. So in these ways, all of the binary codes for the “USTC” letters will be encrypted by this VO_2_ device. In addition, this encryption strategy demonstrated quite high time efficiency if considering the ≈120 µs switching time between the logic “0” and “1” triggered by the electric field (as shown in Figure S4, Supporting Information). The response of this VO_2_ encoder device was much faster than that of the recently reported light encoders prepared by molecular crystal p–n Hetero‐junctions,^[^ ^46^ ^]^ showing excellent time efficiency.

While here it should be noticed that the combinations of voltage and temperature, (*V*, *T*) values, are not one‐to‐one correspondence with the 0/1 logic state, since this two‐channel encryption is unidirectional according to the phase diagram. For the same VO_2_ device, even applied with the same voltage serials, the encryption will be quite different under different temperatures as shown in Figure 6b. Here the voltage serials are set at (8, 16, 10, 12, 12, 12, 14, 15), if the device is set at 30 °C, the encrypted code will be (01000000). While if the temperature is set at other values, the produced codes are quite different. For example, it will be (01000011) at 40 °C and (01111111) at 60 °C, respectively. Therefore, if the decoder intercepts the information of the voltage transmission channel and tries to decode it by forcing the deciphering temperature, the results will be very different at different temperatures. In order to further reduce the probability of information being decoded, we can adopt random values within a reasonable range when transmitting temperature and voltage information according to the phase diagram. With the same phase diagram for the unique VO_2_ device, the receiver can combine the two‐channel serials values to obtain the correct information.

## 3 Conclusion

In summary, we have proposed a multichannel information encryption transmission method based on the insulator‐metal phase transition characteristics of VO_2_ thin film, which can be modulated by various physical fields such as temperature, electric field, and light. Based on the phase diagram of the prepared VO_2_ device, it is possible to divide the encrypted information into multiple physical stimulus channels for encoding transmission, and then read the right information with this hardware‐dependent VO_2_ phase diagram driven by different external stimuli. The multichannel information transmission greatly reduces the risk of information interception and decoding. This work not only achieves a multichannel information encryption strategy, but also opens a new way for the application of phase change materials in data security.

## 4 Experimental Section

### Film Preparation

VO_2_ films were grown on Al_2_O_3_(0001) substrate by rf‐plasma assisted oxide‐MBE instrument working with a base pressure better than 3 × 10^−9^ Torr. During the deposition, the substrate temperature *T*_s_ was maintained at 500 °C, and the growth pressure was maintained at 3.2 × 10^−5^ Torr. Reflection high‐energy electron diffraction (RHEED) was used to monitor the deposition process, and the crystal oscillator (Inficon SQM‐160) was used to measure the film deposition rate. The film thickness is controlled by changing the deposition time. The details of the epitaxial film preparation are reported elsewhere.^[^ ^51^ ^]^

### Material Characterizations

The crystal structures of VO_2_ films were characterized by an X‐ray diffractometer (XRD, model X'Pert MPD, Philips) in the *θ*‐2*θ* scanning mode with Cu K*α* radiation (*λ* = 1.54178 Å). The in situ Raman spectroscopy (LABRAM‐HR) was used to examine the phase transition process as the function of temperature. The excitation source of the spectrometer was 532 nm and the laser power was 5 mW. The microstructure were investigated by Scanning Electron Microscopy (SEM, FESEM SU8220, Hitachi) and (TEM, JEM‐2100PLUS). The surface morphology and structures of VO_2_ film were characterized by atomic force microscopic(AFM, MFP‐3D‐Origin). The X‐ray absorption near‐edge spectroscopy (XANES) was conducted at the XMCD beamline (BL12B) in National synchrotron radiation laboratory (NSRL), Hefei.

### Device Fabrication

Au electrode with various gap was made by UV lithography( Karl Suss, MABA6). Initially, the VO_2_/ Al_2_O_3_ thin film was cleaned by ultrasonic washing with acetone, ethanol, and deionized (DI) water, respectively. Then, the S1813 was spin‐coated on the VO_2_ layer surface (4000 rpm, 60 s) and heated at 180 °C for 90 s on a hot plate. The patterned sample was dipped in AZ300MIF solution for 60 s for developing and subsequently dipped in DI water for 30 s for fixing, followed by the sputter (30 nm Au/Ti) process.

### Electrical Measurement

The resistance as the function of temperature was examined by an electric measurement system (ZJ2810B) with a variable temperature stage (Eurotherm 3504). The Electrical Measurement was carried out in a Semishare four‐probe station with a Keithley 2450 SourceMeter and a coaxial heating chuck.

## Conflict of Interest

The authors declare no conflict of interest.

## Supporting information

Supporting Information

## Data Availability

The data that support the findings of this study are available from the corresponding author upon reasonable request.
