# Supplementary material for: Multiphysical Field Modulated VO2 Device for Information Encryption
Source: Adv Sci (Weinh). 2023 Apr 28;10(21):2300908. doi: 10.1002/advs.202300908 (PMC10375123; doi:10.1002/advs.202300908)
Supplement: Supplementary file 1 — Supporting Information [file ADVS-10-2300908-s001.pdf]

ADVANCED SCIENCE Open Access

Supporting Information

for *Adv. Sci.*, DOI 10.1002/advs.202300908

Multiphysical Field Modulated VO_2 Device for Information Encryption

Shanguang Zhao, Liang Li, Changlong Hu, Bowen Li, Meiling Liu, Jinglin Zhu, Ting Zhou, Weidong Shi and Chongwen Zou**

Supporting Information

Multi-physical Field Modulated VO_2 device for Information Encryption

Shanguang Zhao[1], Liang Li[1], Changlong Hu[1], Bowen Li[1], Meiling Liu[1], Jinglin Zhu[1], Ting Zhou[1], Weidong Shi[2], Chongwen Zou[1]**

[1]National Synchrotron Radiation Laboratory, School of Nuclear Science and Technology, University of Science and Technology of China, Hefei, Anhui 230029, P. R. China

[2] Research Institute of Chemical Defense, Beijing 102205，China

*Corresponding Author: swd1986@mail.ustc.edu.cn , czou@ustc.edu.cn

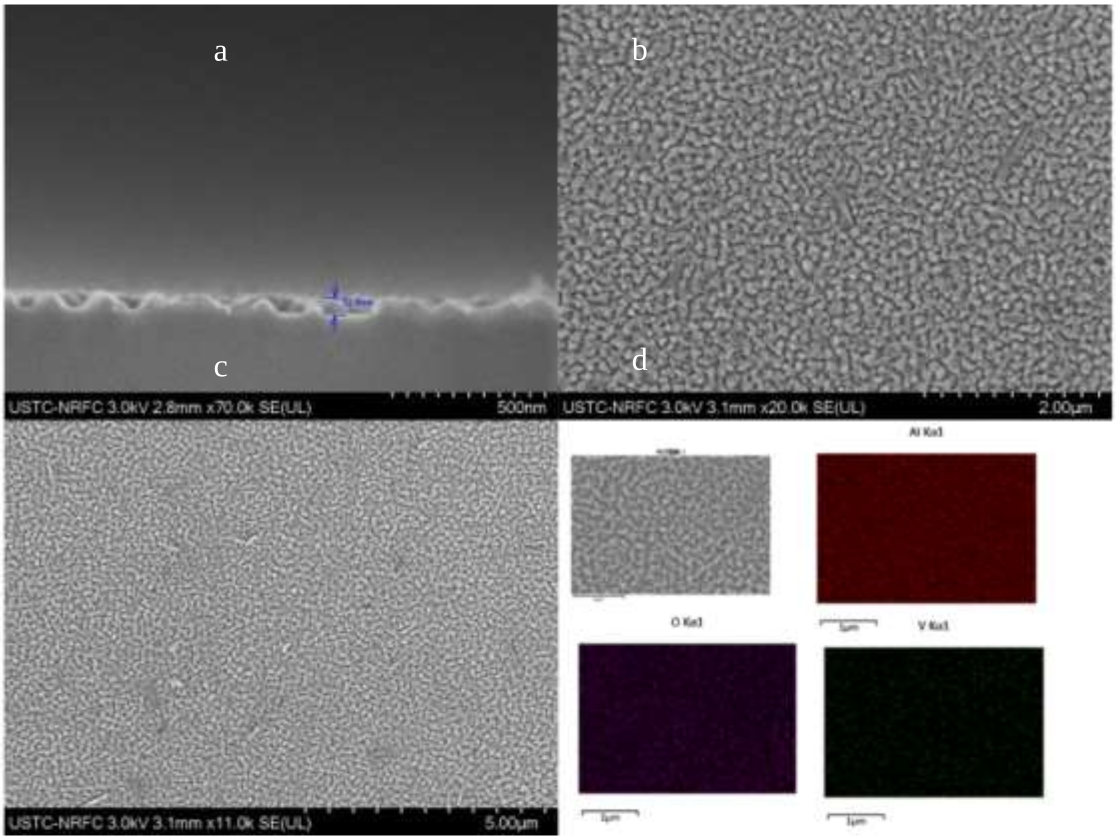

Figure S1. Scanning electron microscopy analysis of VO_2 thin films. a) SEM cross section of VO_2 thin film. b) The surface morphology of VO_2 thin film, scale bar 2μm. c) The surface morphology of VO_2 thin film, scale bar 5μm. d) The EDS mapping for Al, V and O elements, shows a uniform distribution.

.

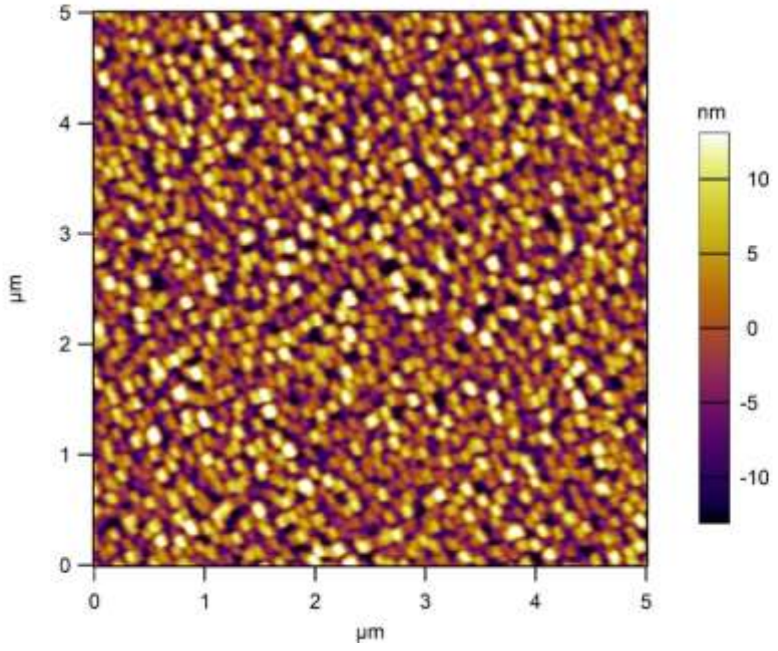

Figure S2. AFM characterization of VO_2 thin films. It shows that the film has a relatively flat surface topography.

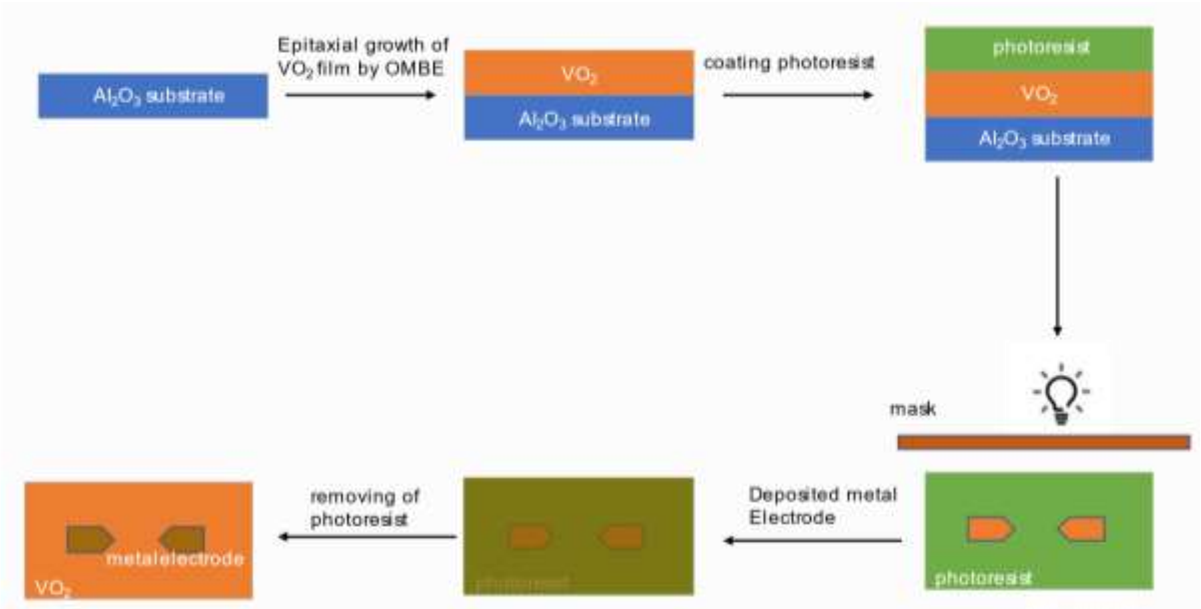

Figure S3. Schematic diagram of steps for making VO_2 devices with different electrode spacing by UV lithography.

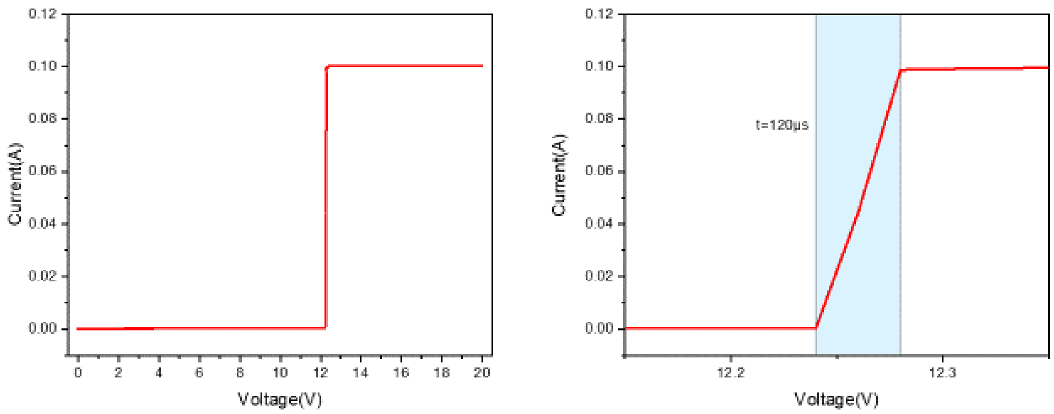

Figure S4. Switching times of the logic “0” and “1” of the VO_2 device during voltage scanning. a) The voltage is scanned from 0 V to 20 V, and the electric field excites the VO_2 metal-insulation phase transition. b) The time of transition from high resistance to low resistance during the phase transition of VO_2 excited by electric field. The temperature is at 50℃.

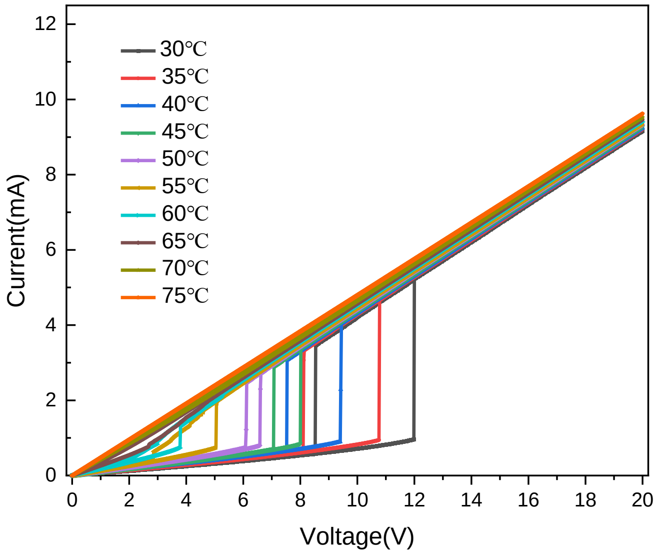

Figure S5. The I -V curves at different temperatures. The temperature regulation of the switching voltage of the VO_2 thin film device, the spacing is 14μm x 2μm.

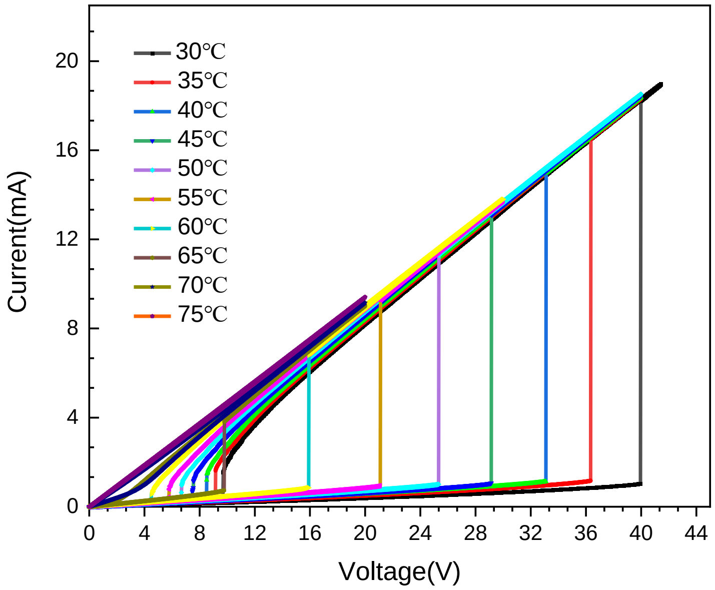

Figure S6. The I -V curves at different temperatures. The temperature regulation of the switching voltage of the VO_2 thin film device, the spacing is 22μm x 10μm.

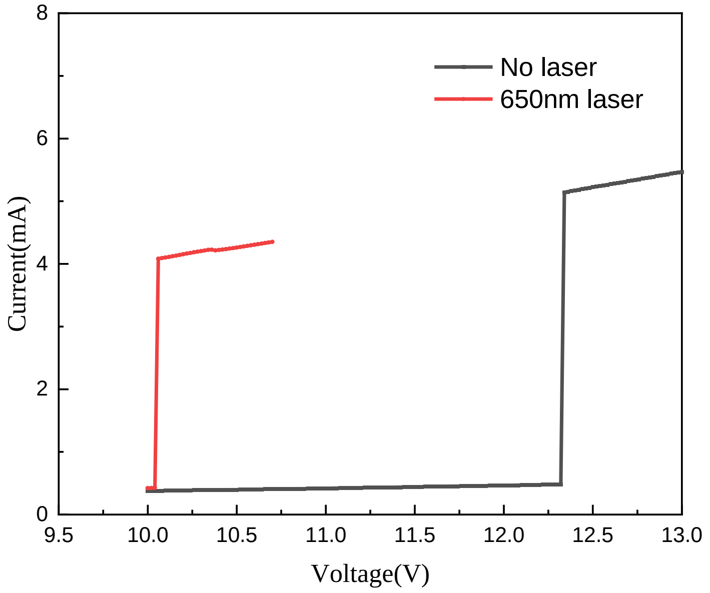

Figure S7. The threshold voltage of VO_2 phase transition switch is regulated by light and electric field. The wavelength of applied light is 650nm.

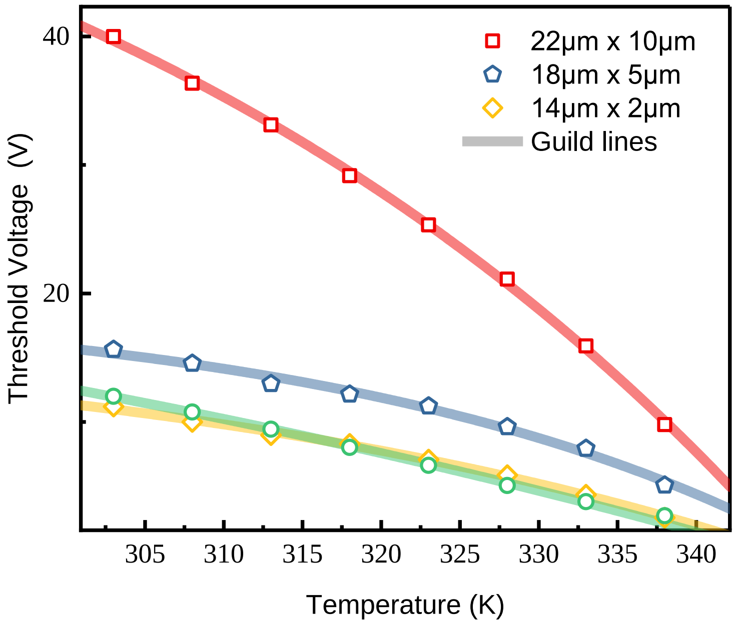

Figure S8. Switching threshold voltages of VO_2 devices with three electrode spacing

widths at different temperatures. The temperature interval of the test was 5K, and the curve was fitted according to the test results.

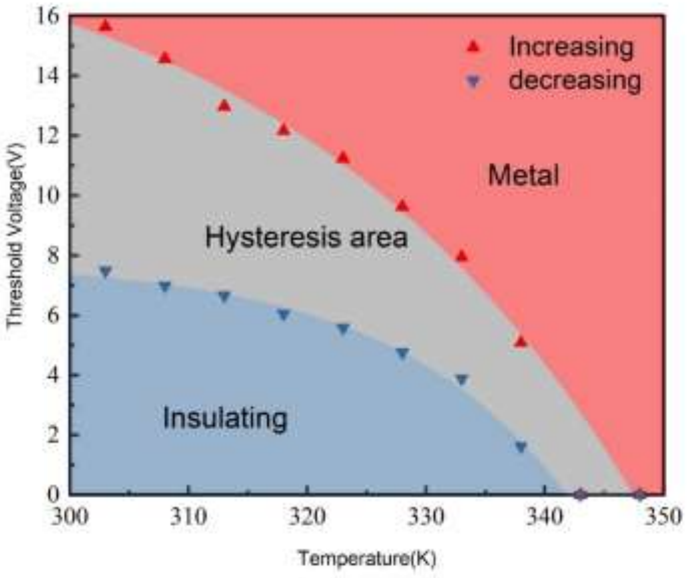

Figure S9. The metal insulation phase diagram of VO_2 device at different temperatures and voltages. The red triangle represents the heating process and the blue triangle represents the cooling process.

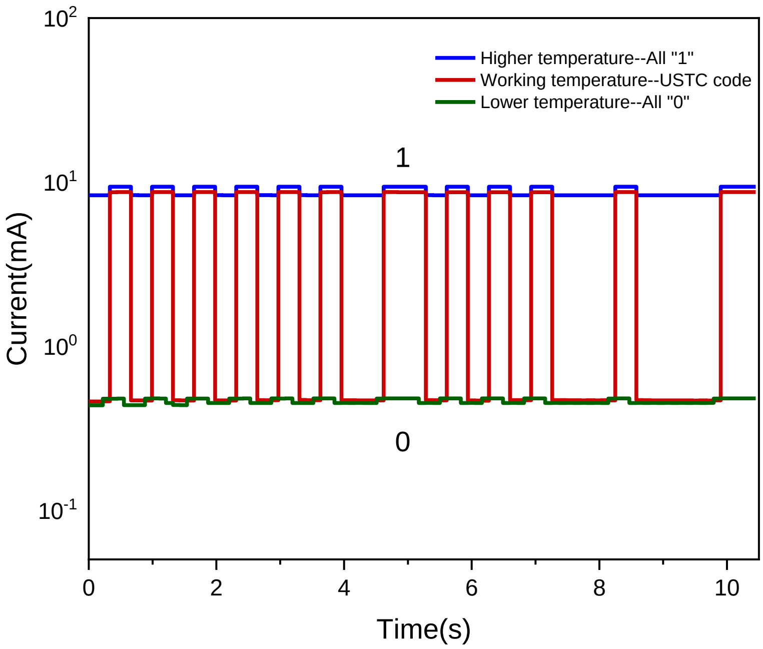

Figure S10. The correct temperature-voltage relationship will get the correct information such as “USTC”, once the temperature is changed, the same voltage will get all “0” or all “1”.
